# Supplementary material for: Adenovirus-Neutralizing and Infection-Promoting Activities Measured in Serum of Human Brain Cancer Patients Treated with Oncolytic Adenovirus Ad5-∆24.RGD
Source: Int J Mol Sci. 2025 Jan 20;26(2):854. doi: 10.3390/ijms26020854 (PMC11765819; doi:10.3390/ijms26020854)
Supplement: Supplementary file 1 [file ijms-26-00854-s001.zip › ijms-3409993-supplementary.pdf]

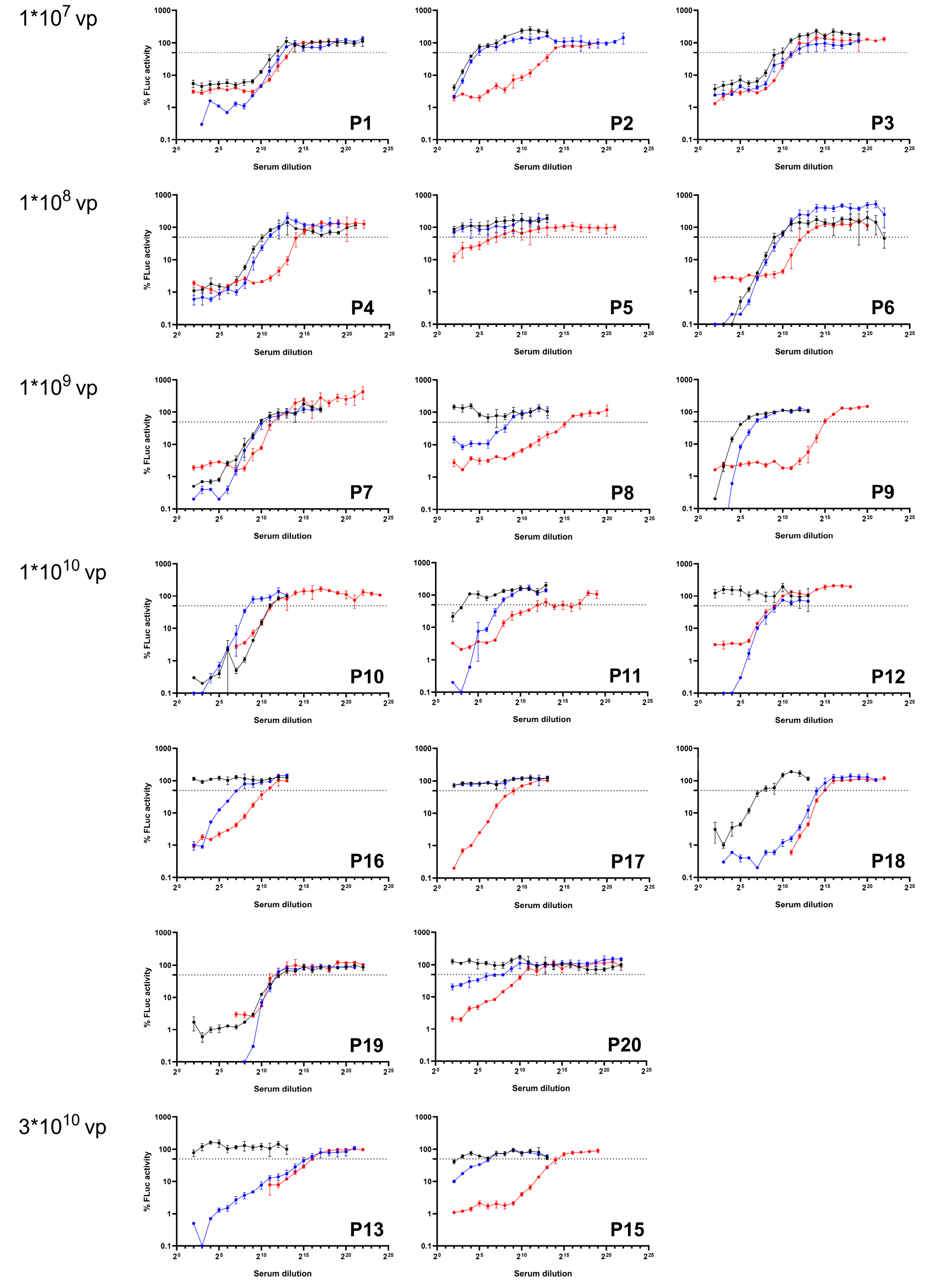

Figure S1. NAb titration experiments using AdLuc. Sera from patients with GBM undergoing treatment with Ad5-Δ24.RGD were 2-fold serially diluted and used to inhibit transduction of A549 cells with AdLuc. Percent transduction compared to controls without serum are calculated. NAb titers were defined as the maximal sample dilution that reduced transduction by at least 50% (broken line). Black lines, pre-treatment sera; blue lines, sera collected 2 weeks after treatment; red lines, sera collected 4 weeks after treatment. Patient numbers are given in the panels; Ad5-Δ24.RGD dose given to each cohort is indicated on the left.

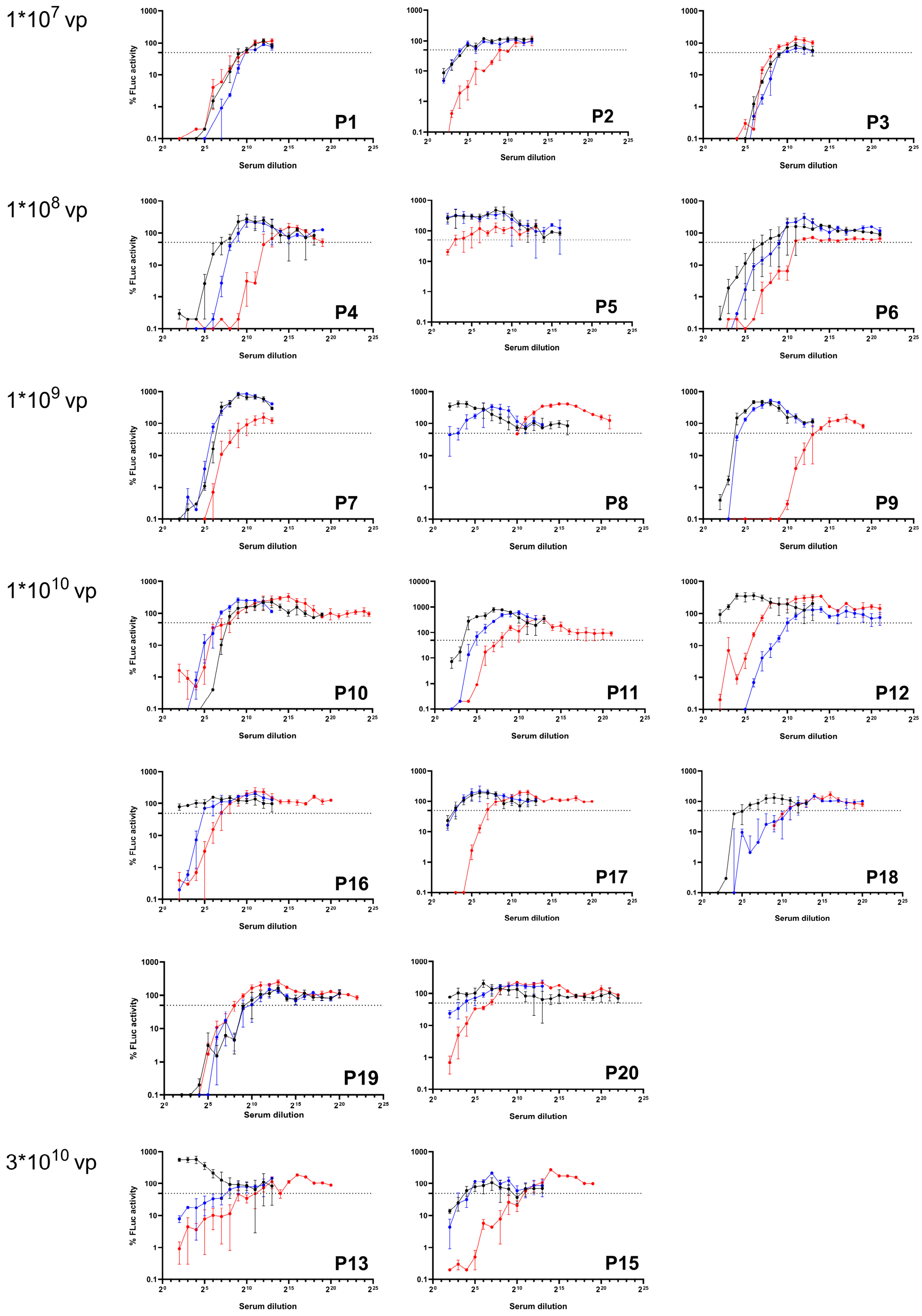

Figure S2. NAb titration experiments using AdLucRGD. Sera from patients with GBM undergoing treatment with Ad5-Δ24.RGD were 2-fold serially diluted and used to inhibit transduction of A549 cells with AdLucRGD. Percent transduction compared to controls without serum are calculated. NAb titers were defined as the maximal sample dilution that reduced transduction by at least 50% (broken line). Black lines, pre-treatment sera; blue lines, sera collected 2 weeks after treatment; red lines, sera collected 4 weeks after treatment. Patient numbers are given in the panels; Ad5-Δ24.RGD dose given to each cohort is indicated on the left.

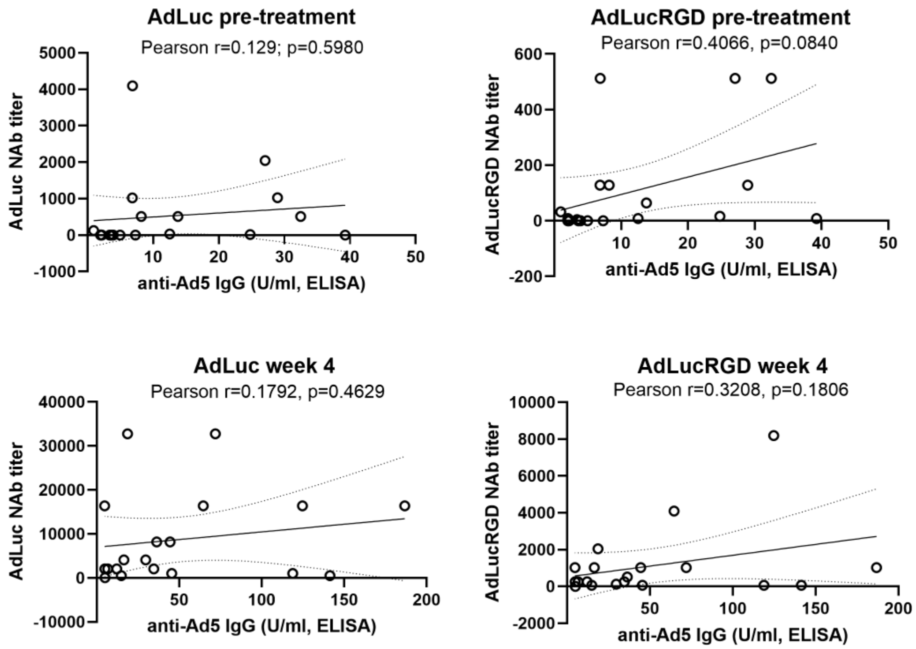

Figure S3. Lack of correlation between total anti-Ad5 IgG titers (van Putten et al., 2022[24]) and AdLuc (left side panels) and AdLucRGD (right side panels) NAb titers, before (upper panels) and 4 weeks after (lower panels) Ad5-Δ24.RGD infusion. Linear regression lines with 95% confidence limits and Pearson correlation coefficients and p-values are given in each panel.

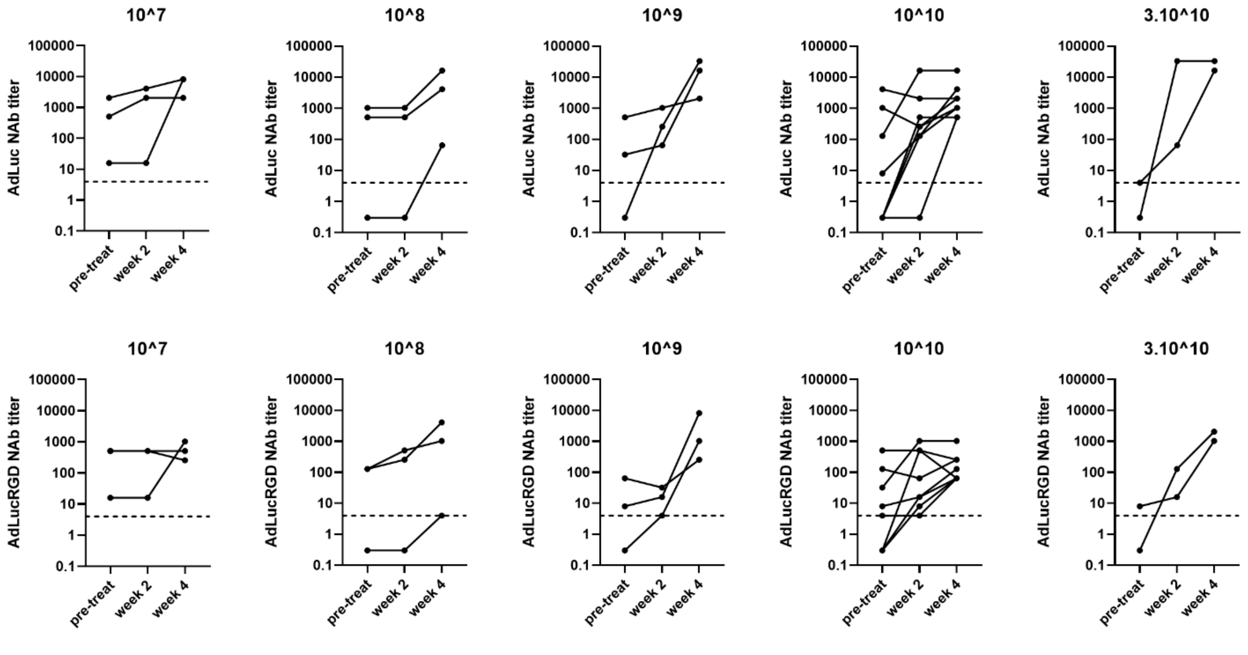

Figure S4. Induction of NAb titers by infusion of Ad5-Δ24.RGD is not dose-dependent. Data of Figure 1B are presented separately for the different dose cohorts. Upper panels, AdLuc NAb titers; lower panels, AdLucRGD NAb titers. Ad5-Δ24.RGD virus copies infused are given above each panel. No correlation between infused copy number and induced NAb titer is observed. Increases in AdLuc and AdLucRGD NAb titers reached within 4 weeks after oncolytic virus infusion were not different between dose groups ($p>0.05$, Kruskall-Wallis test).

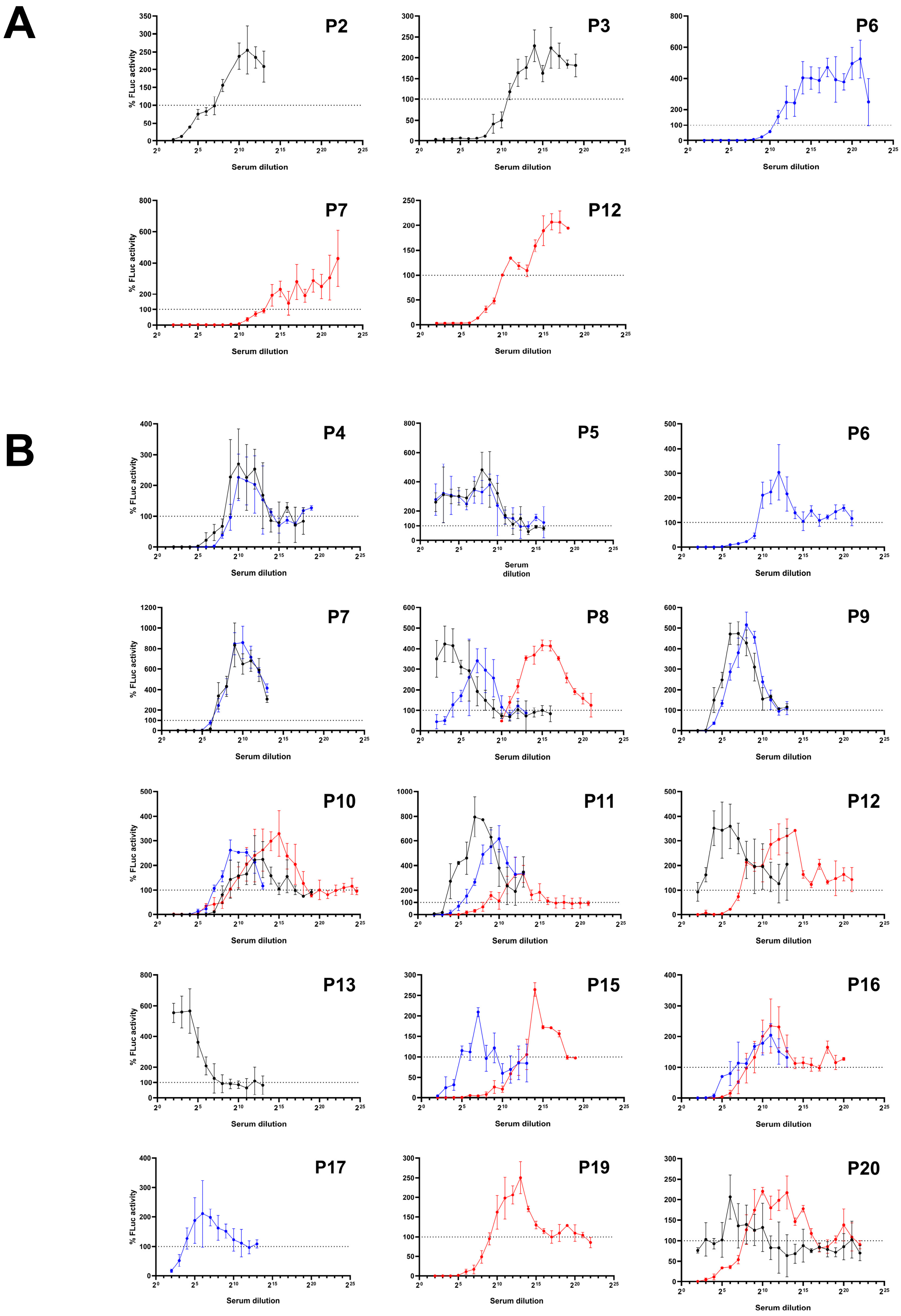

Figure S5. Nab titration experiments revealing serum samples promoting adenovirus vector transduction. **(A)** Sera promoting AdLuc transduction. **(B)** Sera promoting AdLucRGD transduction. Data are the same as shown in Figures S1 and S2, but on a linear scale to appreciate the increased transduction compared to controls without serum (100% reference lines). Black lines, pre-treatment sera; blue lines, sera collected 2 weeks after treatment; red lines, sera collected 4 weeks after treatment. Patient numbers are given in the panels. Only samples that surpass the arbitrary 200% threshold are shown.
